# The Patient Satisfaction Questionnaire Short Form (PSQ-18) as an adaptable, reliable, and validated tool for use in various settings

**DOI:** 10.3402/meo.v18i0.21747

**Published:** 2013-07-23

**Authors:** Anthony Janahan Thayaparan, Eamon Mahdi

**Affiliations:** Department of General Surgery, University Hospital of Wales, Cardiff, UK

Patient satisfaction with the health care provided by doctors is of great significance. Thus, it is important to identify weaknesses in systems to aid improvement through the patient's eyes. This may be done by utilizing the Patient Satisfaction Questionnaire Short Form (PSQ-18), a concise, validated tool that may be applied to various settings, as well as comparing interventions.

Today, we are at an age where health care is scrutinized, not only for the quality of that which we provide but also the satisfaction of those who receive it. Many health care organizations or departments have come under fire due to low patient satisfaction, and this highlights that holistic patient care is integral.

To assess patient satisfaction, there are a variety of questionnaires that may be utilized to identify areas of improvement. However, one such questionnaire ‘the Patient Satisfaction Questionnaire Short Form (PSQ-18)’ (1) has been validated for use in different settings. This was developed through rigorous research and abbreviated from much larger questionnaires (2, 3), maintaining internal consistency and reliability (1). The team behind this Likert scale questionnaire proposed seven dimensions of patient satisfaction directed toward their doctors. These are general satisfaction, technical quality, interpersonal manner, communication, financial aspects, time spent with doctor, and accessibility and convenience.

Each domain is tested through different related questions, which is of substantial benefit when one aims to identify a particular area to improve on. Certainly, general satisfaction has strong correlation with the other domains and thus it is important to improve in all. However, the versatility of a questionnaire allows questions to be tailored to specific domains; one may consider only asking those questions related to communication, to determine whether information has been relayed from physician to patient appropriately and understood well.

We also propose that the PSQ-18 may be used to compare different interventions (such as open and laparoscopic hernia repair), let alone in medical and surgical departments. It has certainly been adapted for use in primary care and the outpatients department (4, 5). The PSQ-18 is a valid, reproducible questionnaire with great potential for use in different settings not to mention well received by patients due to its brevity.
